# International Health

**Published:** 1924-06

**Authors:** 


### INTERNATIONAL HEALTH.

A great Empire Health Congress is about to be held at Wembley where, among many other remarkable things, there is a series of exhibits relating to tropical diseases of such importance that the King directed attention to them in the speech in which he opened the Exhibition. The British Empire has been the pioneer in sanitary enterprise, and it must be at once its duty and its pride to preserve and increase the reputation it has won in that regard. Many other countries, especially the United States, are now exceedingly active in this direction, and we have to look to our laurels. We have taught the elements of sanitary science to half the world, and it is a noble aspiration that its peoples should still turn to us for help. The Wembley Conference should, therefore, have some very valuable results. Without it the Exhibition would have been sadly incomplete. The Empire has to deal with an extraordinarily wide range of diseases among an infinite variety of peoples, and to find, if it can, means of curing and, what is far more important, of preventing them. For these purposes the pooling and co-ordination of the results of scientific investigation are imperative, and we must be as ready to learn as we are to teach.

For these and other reasons we welcome the decision which has just been come to at Geneva by the meeting of Public Health Medical Officers, representing twenty-one nations, to form an International Society of Medical Officers, with its headquarters in Geneva, the historic city of the Red Cross, in which the League of Nations has its home. All the medical men who have taken part in the interchanges organised by the Health Section of the League are to be original members of the Society. A Committee to prepare a Constitution has already been appointed, and it will be complete when our own Society of Medical Officers of Health has selected a representative for Great Britain. The objects which such an organisation must set before itself are as obvious as they are wide. It must needs in the first line seek by every means in its power the advancement of preventive medicine and public health administration in their international aspects. It should form a highly important reservoir of information on public health matters for distribution to its members and, indeed, to all the world, and that it should organise international conferences is inevitable.

There is yet another direction in which this very necessary Society can be of substantial help—it can assist in the promotion of the international activities of the Health Section of the League of Nations. The work of that Section is, as yet, but little known to the world at large, but it is work which covers a vast extent of ground, and has already been of the highest value to the investigator and the statistician. It has been responsible for the visits of a large number of medical officers to and from this and other countries, in which inquiry and comparison have been made easy and thorough. We mention this especially because we regard it as one of the most fruitful of the many efforts in the sanitary direction emanating from Geneva; but it would be easy to compile a long catalogue of the achievements of the League—a catalogue which will, beyond question, become exceedingly formidable as time passes. The International Society of Medical Officers of Health, if it fulfils the intentions of its founders, should be much more than a mere satellite of the Health Section of the League. It should be able to do a great mass of pioneer work, to inform, and even to stimulate, the Section, and, what is not less important, to make better known in every country contributing to its membership exactly what the Section stands for. The League of Nations exists not only for the prevention of war, but for the diminution of industrial strife and the dissemination of knowledge bearing upon physical health. The danger of war between peoples arises, happily, with comparative infrequency, but dangers to health are ever with us. To remove those dangers the League works ceaselessly and often silently, and if the new International Society of Medical Officers can, even in small degree, help and enrich these beneficent activities it will speedily deserve the thanks and support of a humanity from which the peril of physical suffering is, as yet, never far removed.

